# Analysis of the effects of vertical joints on the stability of loess slope

**DOI:** 10.1038/s41598-023-31033-9

**Published:** 2023-03-15

**Authors:** Rong Zhang, Bin Wang

**Affiliations:** 1 Wuhan Railway Vocational College of Technology, Wuhan, 430205 China; 2 China Railway Siyuan Survey and Design Group Co., Ltd., Wuhan, 430063 China

**Keywords:** Geology, Seismology, Civil engineering

## Abstract

Vertical joints with different lengths and *slope distances* (the horizontal distance from joints to slope shoulder) are generated at the top of the loess slope because of the unloading and collapsibility of the loess. The existence of vertical joints is a significant risk factor for loess landslides. This paper applies three methods, including laboratory tests, numerical simulation and theoretical analysis, to analyze the influence of vertical joints on the stability of loess slope. Firstly, the typical failure mode and strength characteristics of the samples containing vertical joints are analyzed through the unconfined compressive strength test and triaxial compression test. Further, the effects of vertical joints with different lengths and the slope distance on the slope safety coefficient and sliding surface position are calculated by finite element numerical analysis. Finally, the formula for calculating the safety coefficient of loess slope with vertical joints is derived. The results show that: the joint bonding degree affects the sample's strength and failure rate. The strength of the sample with open vertical joints is less than that of closed ones, and the failure rate is greater than that of closed ones. When the length and the *slope distances* of the vertical joint are less than 5 m, the influence of vertical joints on the shape and position of the sliding surface will be small. When the length of the vertical joint is more than 5 m and less than 15 m, the safety factor of the slope decreases with the increase in the length of vertical joints. When the length of vertical joints reaches 15 m, and the *slope distance* is 10 m, the deterioration degree of vertical joints on the slope comes up to the peak. Statistical analysis shows that the dominant dip angle of shear fracture at the back edge of the landslide affected by the vertical joint surface is between 45° and 65°. The research results can be used for rapid calculation of the safety coefficient and rapid evaluation of slope stability of loess slope with vertical joints.

## Introduction

Loess is a Quaternary sedimentary soil. The loess stratum is non-homogeneous and anisotropic from top to bottom, covering various paleosol layers, calcareous nodules, pores (including sink holes), fissures, and joints. Among them, loess joints are ubiquitous in the loess stratum, especially vertical joints, which are one of the main features of loess^1–3^. In previous studies on the genesis mechanism of loess joints, except for the cause of tectonic movements, it is believed that vertical joints in slopes and underground spaces are generated under the influence of three factors, namely, collapsibility of loess^1^, the horizontal tensile stresses caused by the overlying load after excavation^2,3^, and the unsaturated properties of loess^4^. Loess joints can be divided into open joints and closed joints. Open joints are mostly tensioned vertical joints produced by rainfall, weathering, and wet sinking^5^. Closed joints are common after the excavation of loess, with irregular distribution of directionality and length. Closed joints distribute deeper, which are more widely found in Q2 loess in field sampling. The survey found many vertical joints and fissures in the soil at the back edge of the slope, the edge of loess tableland, and the soil near the free surface^4–6^. Vertical joints influence the back edge of loess landslides, and the steep ridges are considered the tensile fracture surfaces with vertical joints^7–9^. The existence of loess joints, on the one hand, is equivalent to the division of the slope, reducing the integrity of the slope and increasing the anisotropy of the loess^10^; on the other hand, the loess area is dry and less of rainfall all year round, and most of the loess is usually in a low moisture content state. The vertical joints become the dominant seepage surface^11^, allowing rainfall to infiltrate through the shallow surface of the loess to the deep loess^12,13^. At the same time, the joint’s shear strength and tensile strength decrease, and the self-weight of the soil increases. So the stability of the slope reduces, which can further lead to the instability of the loess slope with substantial wetting^14,15^. Thirdly, the closed vertical joints will further expand into open joints and ground cracks under unloading, wetting, and rainfall infiltration^16^, increasing the probability of landslides. Therefore, the vertical joints in loess are essential factors affecting the stability of loess slopes. It is significant to study the stability of loess slopes with vertical joints.

The research on the stability of slope with vertical joints mainly focuses on numerical simulation^17^, field survey and machine learning techniques^18^. For example, Mao^4^ studies the influence of the length of vertical joints and horizontal distance from the slope surface on the stability of slopes using the extended finite element method. He considers that the influence of vertical joints on slopes is most outstanding in the range of 5–9 m from the slope surface, and the effect is negligible when the depth exceeds 12 m. Yang^19^ and Ma^9^ investigate the loess landslide of the South Plateau of Jingyang and propose that the distance of collapsible vertical fissures from the loess edge is 15–30 m. The distance of unloading vertical fissures from the loess edge is no more than 7 m. Actually, there are not only vertical joints in the original loess but also many diagonal joints with different dip angles. Sun^20^ finds the mechanical properties of original fractured loess with four different dip angles from 0° to 60° by triaxial compression test. She concludes that the fracture surface with original joints can influence the soil failure surface. The standard evaluation index of soil slope stability is the safety factor, which can be calculated by the limit equilibrium method, strength reduction method and other methods. Currently, the commonly used calculation software for numerical simulation includes the finite element, finite difference, and discrete element. At present, the commonly used numerical simulation calculation software are finite element, finite difference, discrete element and other calculation software. Table 1 lists the standard calculation methods for slope stability and the advantages and disadvantages of each method. The finite element calculation software can use the contact unit to simulate the joint surface, which is a typical method to simulate the discontinuous medium, and also can calculate the safety factor of slope by the strength reduction method. Therefore, the finite element method is applicable to the stability calculation of local discontinuous slopes. This paper uses Midas GTS finite element calculation software to calculate the coefficient of safety for loess slopes with different lengths and *slope distances*.

**Table 1:** Comparative analysis of the advantages and disadvantages of two typical slope stability calculation methods^21–23^.

Calculation method	Limit equilibrium method	Strength reduction method
Core principles	Calculation of slope stability coefficients using the static equilibrium equation based on the slice method	Different reduced coefficients are used to change the material cohesion and internal friction angle parameters, and the reduced coefficient at critical damage is defined as the safety factor of the slope
Advantages	Short calculation time, either by hand or numerically with the aid of a computer	Long calculation time, suitable for non-linear, non-homogeneous and complex slope stability numerical calculation
Disadvantages	Sliding surfaces need to be assumed in advance	No prior assumptions about the position and form of the slip surface are required

Therefore, in the study of slope stability under the influence of vertical joints, whether the existence of vertical joints will influence the location and failure mode of the sliding surface of the slope and the influence range should be the key questions to be answered in the research of the influence of vertical joints on slope stability. The exploration of vertical joints is based on investigating the fracture characteristics of the samples containing vertical joints through soil mechanical experiments, especially the direction of the fracture surface. It needs to establish a numerical model of vertical and diagonal joints inside the slope simultaneously and calculate the influence of the joint surface on the position and failure mode of the sliding surface of the slope.

**Figure 1:**
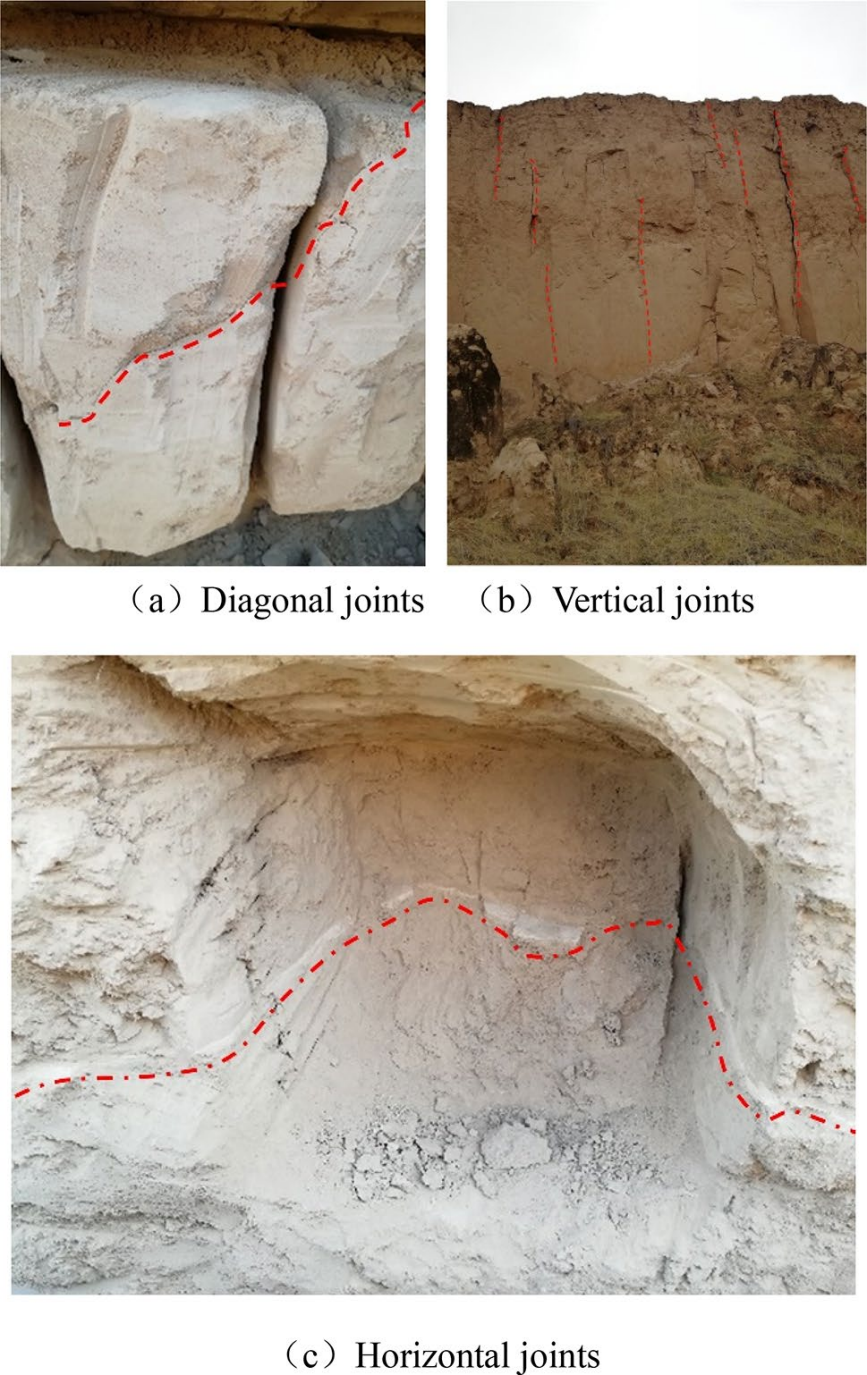
Extensive development of joints in loess.

This paper explores the failure characteristics and mechanical properties of the jointed loess samples through triaxial tests, based on which the finite element model is established, and the length effect, the impact of horizontal distance from joints to slope shoulder (subsequently referred to as the *slope distance*), and inclination effect of vertical joints are calculated. The safety factor of the slope is calculated by using the finite element strength reduction method. Finally, the formula for calculating the stability coefficient of slope with vertical joints is given through theoretical derivation. Figure 1 is taken by the author of the paper during field sampling in the loess area of western China, and it illustrates three typical joints of the loess in western China (diagonal, vertical, and horizontal).

## Material and sample preparation method of soil mechanical experiments

It is significant to research the mechanical change law of vertical joint samples to understand the evolution process of tensile fracture at the back edge of the slope and the influence of vertical joints on the sliding surface of the slope. The loess used for the test was taken from Yongguang Village, Minxian County, Gansu Province, China, at the top of a large landslide caused by the 2013 Minxian—6.6 earthquake, with a sampling depth of 1 m. The test results show that the loess’s natural moisture content is 6.3%, plastic limit is 17.83%, liquid limit is 23.33%, the optimum moisture content is 16.5%, and maximum dry density is 1.68 g/cm^3^.

**Figure 2:**
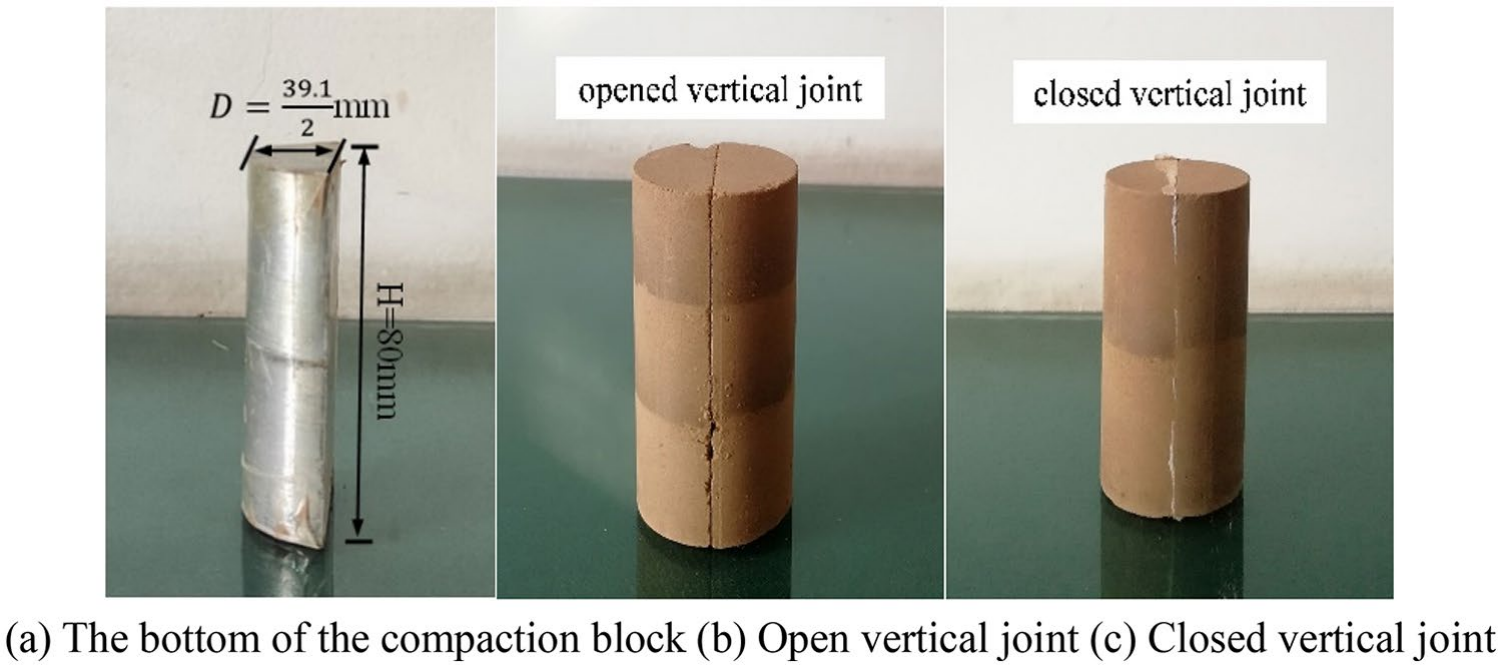
Two kinds of vertical joint samples with different bonding degrees.

Vertical joints can be divided into open vertical joints and closed vertical joints according to the degree of adhesion of the joint surface. To simulate the mechanical properties of the joint samples with different degrees of opening, the writer modifies the bottom of the traditional compaction block of sample preparation and produces two types of vertical joint samples, closed and open. The open joint samples are fabricated with the compaction block in Fig. 2a. The samples are made in two parts and compacted in layers. The two parts are spliced to form the ones in the open vertical joint test shown in Fig. 2b. The closed joint samples are fabricated by the method in literature^24^, where 1/2 sample is made first. The joint surface is scraped, and additional isolation material is added. Then the samples are placed back into the compaction barrel to form the closed vertical joints in Fig. 2c with the cooperation of the compaction block in Fig. 2a.

## Strength and failure characteristics of vertical joints

The water content of samples w = 10.0%, the density ρ = 1.69 g/cm^3^, and the degree of compaction is 91.5%. The samples' typical failure mode and stress–strain relationship are discussed as follows.

**Figure 3:**
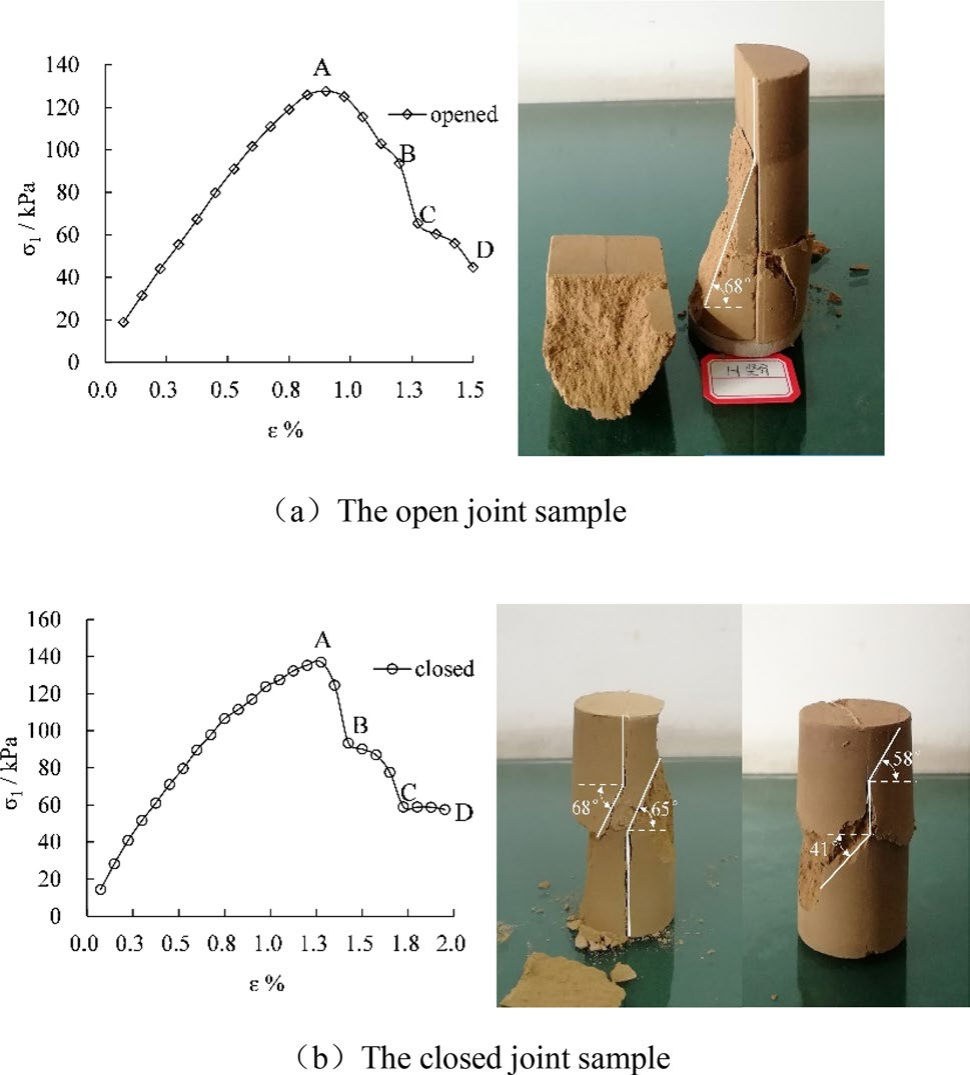
The stress–strain curve of vertical joint samples by unconfined compressive strength test and typical failure characteristics.

Figure 3 exhibits the typical stress–strain curves of the unconfined compressive strength test for the two joint samples and the corresponding failure diagram.

The strength characteristics of the two different joint samples demonstrate that the strength of the closed type is slightly greater than that of the open type, and the strength of the closed vertical joint samples and the open vertical joint samples is 15.7% and 21.5% lower than that of the samples without joints respectively. From the failure rate of the samples investigated by strain corresponding to the peak strength, it is clear that the failure rate of the open joint sample is greater than that of the closed joint sample.

The reasons for the strength and failure differences are analyzed in terms of the samples' failure process and failure mode.

The comparative research on the failure of closed and open joint samples testifies that shear fracture occurs first in closed joint samples. Due to the existence of vertical joints, the vertical joint surface is partially open and connects with the failure surface when the shear fracture surface develops to the joints. The dip angle of the shear fracture surface β = 41°–68°. The failure of open joint samples is similar to that of closed ones. The shear fracture occurs first, while because the joints are fully open, the fracture surface completely passes through the joint surface. The dip angle of shear fracture surface β = 68°. Since the closed joint sample has a particular adhesive strength at the joint surface, its uniformity and integrity are better than that of the open joint sample, and the strength is slightly greater than that of the open one.

The post-peak stress–strain curve in Fig. 3 can be divided into three stages: the initial shear fracture stage of the sample occurs in the A–B section; the multiple stress drops in the B–C and C–D sections indicate that the test experienced multiple tensile brittle failures after the peak^25^, which can correspond to various shear fracture surfaces of the samples, and tension cut-through of the vertical joint surfaces.

The strength and deformation, and failure characteristics of the vertical joints are summarized as follows: closed vertical joints are slightly higher than open vertical joints in terms of strength; the failure rate of open vertical joints is more rapid than that of the closed vertical joints; in terms of failure characteristics, both types of joints undergo three stages of "shear fracture—the cut-through of the shear fracture surface and vertical joint surface—residual deformation". Closed vertical joints can change into open vertical joints under specific external forces, which verifies the view of Feng^5^ and others on the development mechanism of vertical joints in loess.

Three kinds of confining pressures, 50 kpa, 100 kpa, and 150 kpa, are used in the triaxial compressive test. The stress–strain curves and typical failure states of samples containing closed vertical joints under the three confining pressure conditions are summarized as follows.

**Figure 4:**
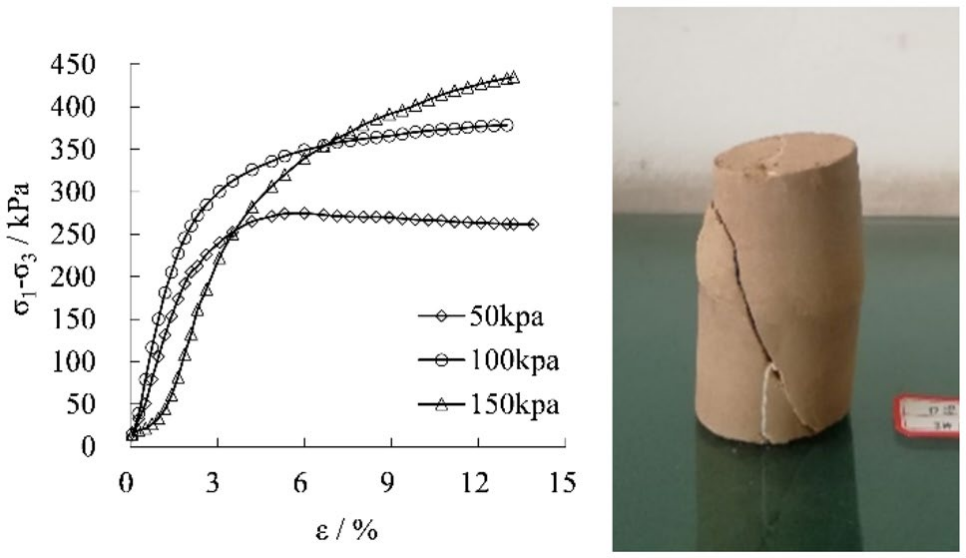
The stress–strain curve of vertical joint samples by triaxial compression test and typical failure characteristics.

Figure 4 manifests that the stress–strain curve gradually displays the strain-hardening feature with the rise of the confining pressure. The failure of samples also shows the typical shear fracture characteristics, indicating that the vertical joint's influence on the strength and failure pattern gradually decreases. Similarly, it can be presumed that the effects of vertical joints on slope stability will decrease progressively with the increase of lateral restraint and burial depth on the slope.

## Numerical analysis of the stability of the joint slope

The vertical joint's length is defined as* h*, and the distance from the vertical joint to the slope shoulder is defined as *d*. Firstly, this paper sets up a slope model with vertical joints of different lengths and the *slope distances* to study the distribution of the leading plastic zone and the change of safety coefficient in the limit state of the slope containing vertical joints only. On this basis, the slope models with vertical and diagonal joints are established to analyze slope stability further. Meanwhile, a slope model without joints is a reference for comparison. Midas GTS finite element software is used for numerical calculation. The model is a two-dimensional model. The bottom of the model is a fixed boundary and the side is a unidirectional constraint boundary. The minimum grid size is 0.5 m, and the calculation method of safety factor is strength reduction method.

### Length effect of vertical joints and slope distance effect

The model's height is 40 m, and the slope angle is 45°. The length of vertical joints and the *slope distance* are 5 m, 10 m, 15 m, and 20 m, respectively. "Interface element" of finite element simulates the joint surface. The joint surface is simulated by lessening the cohesion of the contact surface and expanding the ratio of standard stiffness to tangential stiffness. The model investigates the influence of vertical joint size and the distance of joints from the slope shoulder on the slope's stability, and Kn/Ks = 100. The cohesion and internal friction angle of loess are measured by triaxial test, and the cohesion and internal friction angle of joint surface are calculated according to Formulas (1) and (2)^26^

1 $${c}_{inter}={R}_{inter}c$$

2 $$\mathrm{tan}{\delta }_{inter}={R}_{inter}tan\varphi$$

where c and φ are the cohesion and internal friction angle of the soil, respectively, R_inter_ is the strength reduction coefficient of the joint surface, and c_inter_ and φ_inter_ are the cohesion and internal friction angle of the joint surface after reduction, according to the literature^27^, the discount factor R_inter_ is taken as 0.6. Table 2 lists the mechanical parameters of the soil slope and the joint surface.

**Table 2:** Mechanical parameters of the models.

Designation	γ (kPa)	c (kPa)	Φ
Loess	18	100	26
Joint surface	–	60	16.3

**Figure 5:**
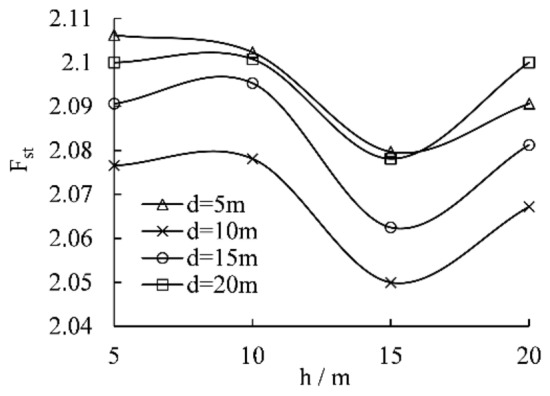
Relationship between safety factor and the length of vertical joints.

The stability of the slope is calculated by the strength reduction method. By changing the vertical joint length and the distance from the shoulder of the slope, the variation curve of the safety coefficient with the joint length in the slope model containing a single vertical joint under self-weight is calculated as shown in Fig. 5.

As demonstrated in Fig. 5, the safety factor of the slope* F*_*st*_ shows a changing trend of decreasing and then increasing with the growth of the joint's slope distance *d* and reaches the valley value at d = 10 m and h = 15 m; that is, the slope reaches the maximum deterioration with the vertical joint of 15 m at a distance of 10 m from the slope shoulder. Joints with an inclination of about 80° were found at the back edge of the landslide in Zaoling Township, Xiangning County, Linfen City, Shanxi Province, China, on March 15, 2019. The original slope of this landslide is 45°, and the height is 70 m, which is a typical water-induced landslide. The vertical joints play the role of a water-conducting channel in the process of landslide generation. The horizontal distance between the leading edge and the back edge of the landslide is 15.9 m^28^. Compared with the slope of 45° and 50 m height calculated in this paper, the height of the landslide in Xiangning is high. So its potential energy is high, and the range of the landslide is slightly larger than 10 m.

**Figure 6:**
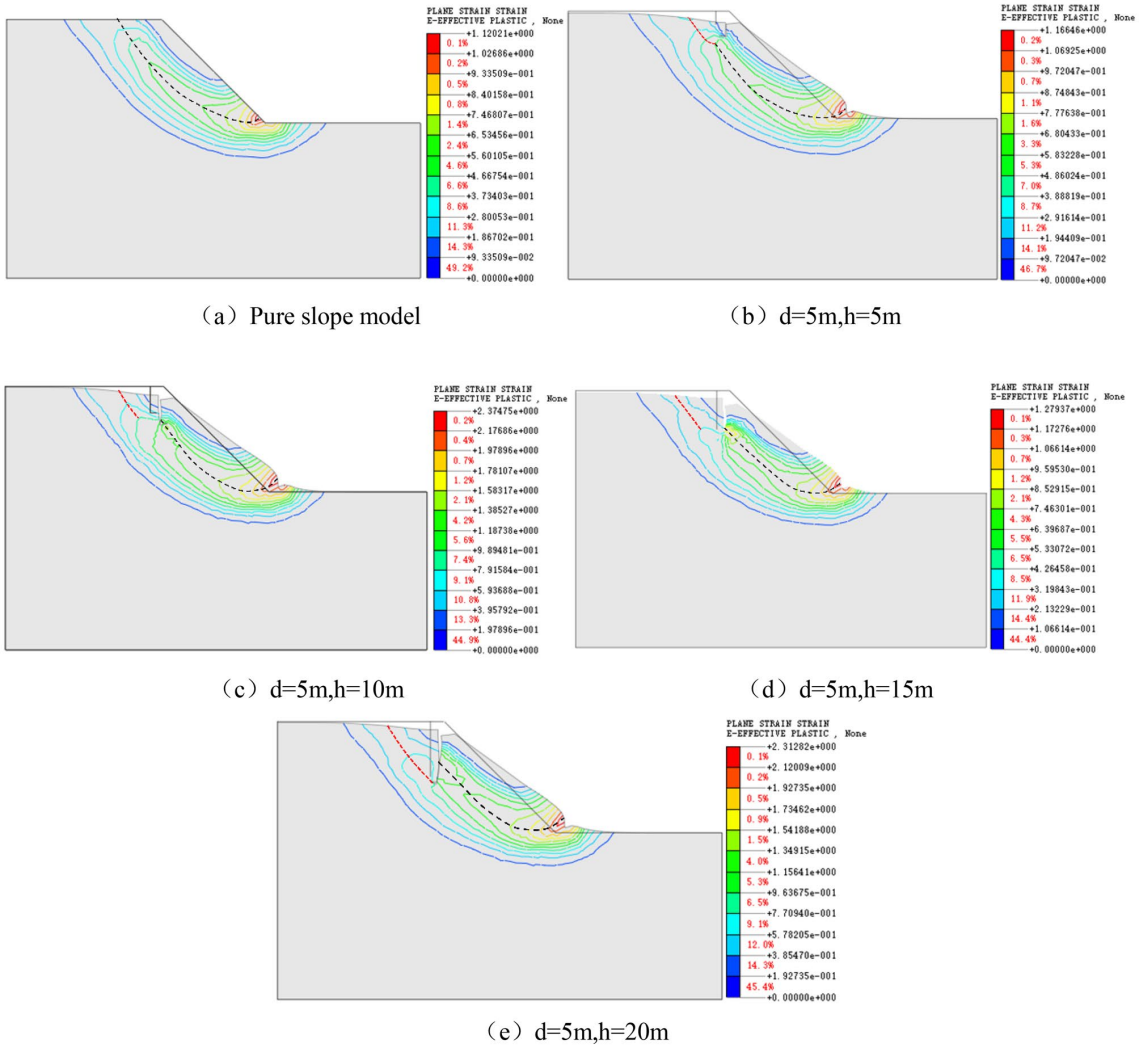
Cloud chart of the plastic zone and sliding surface with vertical joints d = 5 m and h = 5–20 m.

**Figure 7:**
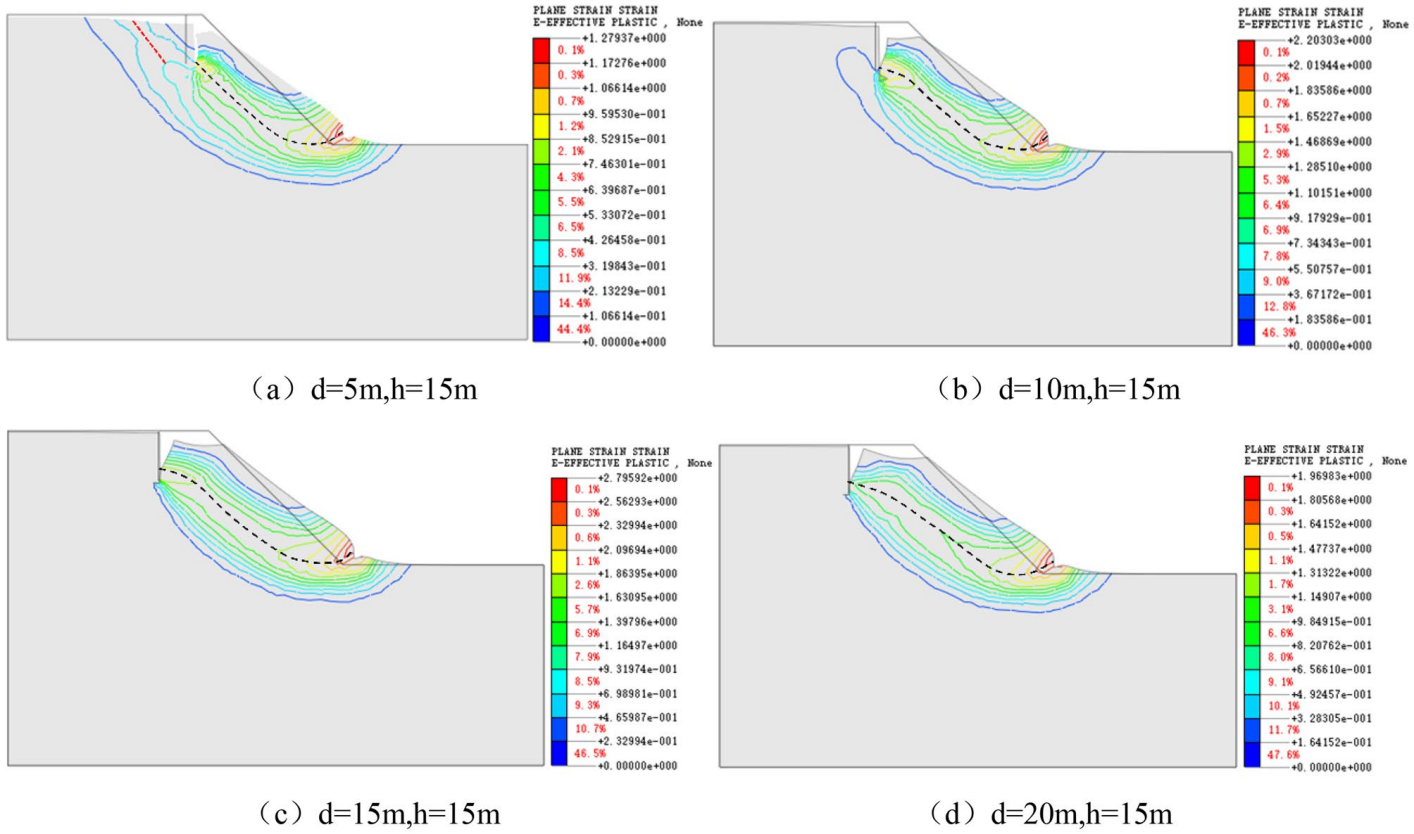
Cloud chart of the plastic zone and sliding surface with vertical joints h = 15 m and d = 5–20 m.

In the cloud chart with the contour line of the plastic zone with a limit state, peak points of the central contour line are connected and defined as the sliding surface of the slope. This paper takes the models with d = 5 m and h = 5–20 m as examples to analyze the influence of the joint length on the change of cloud chart of the plastic zone and the sliding surface of the slope (Fig. 6); taking the model with h = 15 m and d = 10–20 m as examples, the influence of the change of the joint’s slope distance on the cloud chart of the plastic zone and the sliding surface is analyzed (Fig. 7).

As displayed in Fig. 6, firstly, the cloud chart with the contour line of the plastic zone at the bottom of the vertical joints are denser, which indicates that the stress concentration phenomenon occurs here and the actual slope is subjected to more considerable tensile stresses; secondly, the failure surface of the slope consists of two parts, the tensile zone at the top of the slope (red dashed line) and the tensile zone at the bottom of vertical joints + shear zone at the bottom of vertical joints (black dashed line); thirdly, with the increase of the length of vertical joints, the influence of vertical joints on the overall sliding surface of the slope gradually improves. When the length of vertical joints is h = 5 m, the sliding surface of the slope is similar to the pure slope model without joints, and the sliding surface does not pass through the bottom of vertical joints. When the length of vertical joints is h = 10–15 m, the failure of the slope starts from the bottom of vertical joints to shear out at the foot of the slope. When the length of vertical joints is h = 20 m, the sliding surface of the slope no longer starts from the bottom of the vertical joints but from the middle and lower part of the vertical joints, which is consistent with the failure results of the soil mechanical experiments (Fig. 4), indicating that there is an extreme value of the influence of the length of vertical joints on the sliding surface. The stress concentration at the bottom of vertical joints reaches the peak when the length of the vertical joint h = 5 m. So the length of the vertical joint h = 15 m has the most significant influence on the stability of the slope, which is identical to the research result that the safety factor is the smallest at h = 15 m, as shown in Fig. 5.

Figure 7 exhibits that the sliding range of the slope increases gradually with the enhancement of the *slope distance* of vertical joints. When the *slope distance* of vertical joints is d = 10 m, the sliding surface of the slope starts from the bottom of vertical joints and ends at the foot of the slope, and the sliding surface includes the whole vertical joint surface. When the *slope distance* of vertical joints exceeds 10 m, the sliding surface no longer starts from the bottom of vertical joints but the middle and lower part of vertical joints, which means that when d = 10 m and h = 15 m, the deterioration of slope caused by vertical joints reaches the maximum.

### Shear fracture characteristics of the back edge of sliding surface of the slope with vertical joints

Soil mechanical experiments and numerical simulations both manifest that the failure mode of soil with vertical joints is "tension + shear fracture". The soil mechanical experiments show that sliding failure can occur along the joints when the dip angle of diagonal joints is β = 45°–70°, and the dip angle of shear fracture of vertical joints is 41°–68°. Some models are designed to verify the reasonableness of the unit study results of the soil mechanical experiments and further investigate the characteristics of unstable sliding motion of slopes containing vertical joints. Meanwhile, the dominant failure inclination of the back edge of the slope can reveal the disaster-causing mechanism. The model parameters are presented below: lengths of vertical joints h = 15 m; the *slope distance* d = 10 m; the diagonal joints with the length of l = 11.5 m are added at the bottom of the vertical joint, and the included angles of the diagonal joints with horizontal direction *β* are 30°, 45°, 57°, 65° respectively.

**Figure 8:**
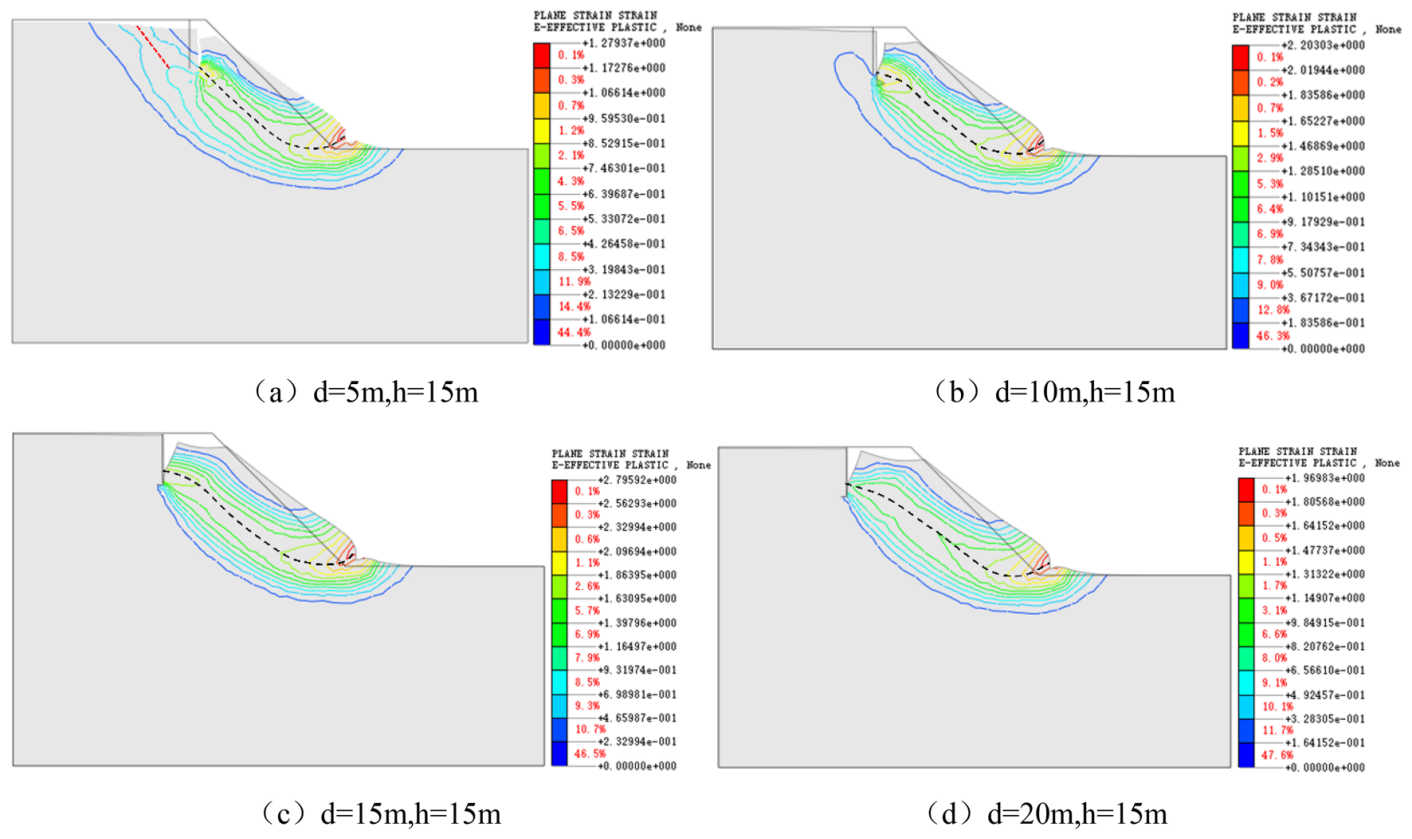
Cloud chart of the plastic zone and sliding surface at the back edge of landslide with dip angle β = 30°–65°.

Figure 8 illustrates the main sliding surface of each model and the cloud chart with the contour line of the plastic zone.

**Figure 9:**
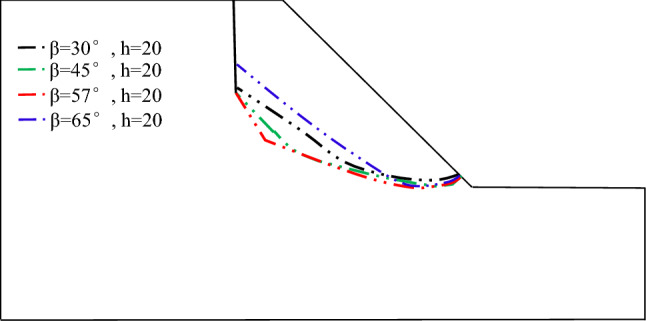
Main sliding surface of slopes with different dip angles of diagonal joints.

The sliding surfaces in Fig. 8 are summarized as shown in Fig. 9.

The stress concentration points of each model in Fig. 9 are marked with black circles. The stress concentration points are mainly concentrated at the bottom of vertical joints, the bottom of diagonal joints, and the foot of the slope. So there is a possibility of pulling cracks along vertical joints and sliding failure along diagonal joints. The change of the main sliding surface with the dip angle of diagonal joints reveals that when the dip angle of diagonal joints β = 45° and 57°, the plastic strain at the slope toe and the bottom of diagonal joints is obvious. It means that the slope is prone to sliding failure along the diagonal joint surface; therefore, the back edge of the landslide of the sliding surface containing vertical joints has a dominant shear fracture inclination of 45°–57°, with an average value of 51°, which is close to the soil mechanical experiments of 41°–68°, with the average value of 54.5°. This result is close to the statistical result of the soil mechanical experiments of 41°–68° with a mean value of 54.5°, which shows the reasonableness of numerical calculation results and the validity of testing results.

**Figure 10:**
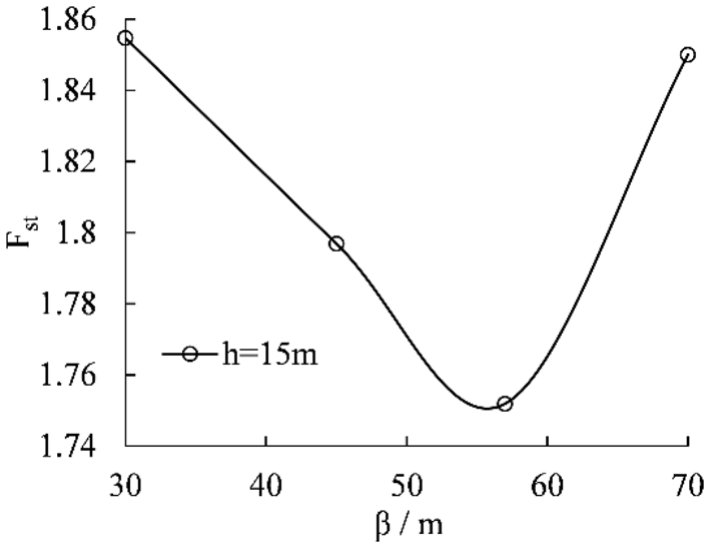
The variation curve of slope safety factor with the dip angle and length of the joint.

The statistical analysis of the safety coefficients of each model is carried out, as shown in Fig. 10. The safety coefficient of the slope is the lowest when β = 57°, which is consistent with the conclusion obtained from the distribution of plastic zone and the change of sliding surface.

## Exploration of the mechanical model for the instability of loess landslide with vertical joints

**Figure 11:**
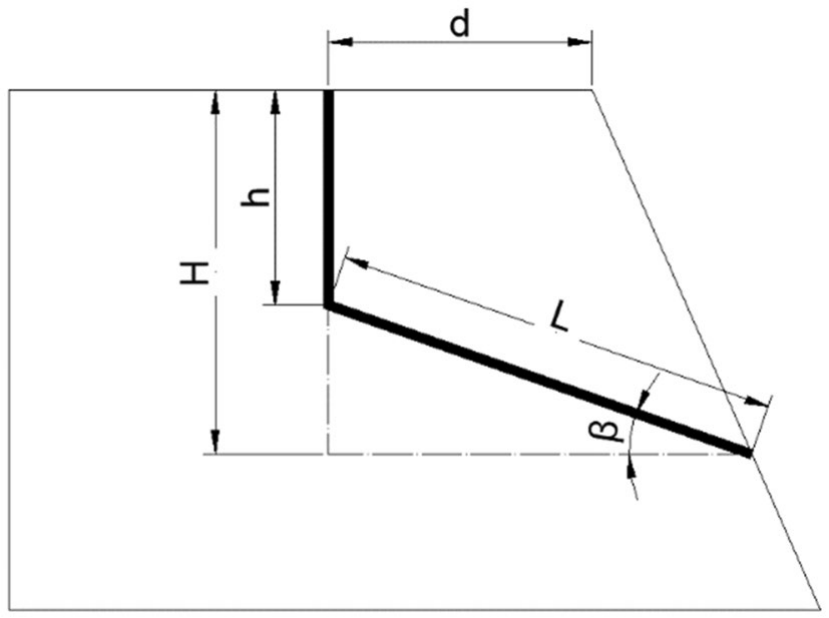
Schematic diagram of analytical calculation.

When the slope containing vertical joints becomes unstable, to investigate the relationship between the slope safety factor and the length of vertical joints, the dip angle of the potential sliding surface, and the slope length, a schematic diagram of the slope model with vertical joints are created as depicted in Fig. 11. Define that the length of vertical joints is *h*, the horizontal distance from the slope surface is *d*, the dip angle of shear fracture at the back edge of the landslide is *β*, the circular sliding surface is simplified as a straight line, and its length is *L*.

Assume that the tensile strength of the vertical joint surface is *σ*_*h*_, and the shear strength of the shear fracture surface is shown below.

3 $$\tau =\sigma tan\varphi +{c}_{l}$$

where *φ* is the internal friction angle of the shear fracture surface and *c*_*1*_ is the cohesive force of the shear fracture surface.

The total height *H* of the trapezoid above the slip surface can be converted to the following equation.

4 $$H=h+Lsin\beta$$

The weight of the trapezoid above the combination of the slip surface is expressed below:

5 $$\begin{aligned} {\text{w}} & = \frac{1}{2}\gamma H\left( {Lcos\beta + d} \right) - \frac{1}{2}\gamma Lcos\beta \cdot Lsin\beta \\ & = \frac{1}{2}\gamma H\left( {Lcos\beta + d} \right) - \frac{1}{4}\gamma L^{2} sin2\beta \\ & = \frac{1}{2}\gamma (Lhcos\beta + Ldsin\beta + hd) \\ \end{aligned}$$

According to the definition of safety factors:

6 $${K}_{s}=\frac{{F}_{t}}{{F}_{s}}$$

Sliding force

7 $${F}_{s}=\mathrm{wsin\beta }-{\sigma }_{h}\mathrm{cos\beta }$$

Anti-sliding force

8 $${F}_{t}=(\mathrm{wcos\beta }+{\sigma }_{h}\mathrm{sin\beta })\mathrm{tan\varphi }+{c}_{l}$$

Substitute (5), (6) into (4) to get the following equation.

9 $$\begin{aligned} K_{s} & = \frac{{F_{t} }}{{F_{s} }} = \frac{{\left( {{\text{wcos}}\beta + \sigma _{h} {\text{sin}}\beta } \right){\text{tan}}\varphi + c_{l} }}{{{\text{wsin}}\beta - \sigma _{h} {\text{cos}}\beta }} \\ & = \frac{{w + \sigma _{h} tan\beta }}{{wtan\beta - \sigma _{h} }}tan\varphi + \frac{{c_{l} }}{{wsin\beta - \sigma _{h} cos\beta }} \\ \end{aligned}$$

If the vertical joint surface is an open type, or the tensile strength *σ*_*h*_ is minimal, then *K*_*s*_ is written as:

10 $${K}_{s}=cot\beta tan\varphi +\frac{{c}_{l}}{wsin\beta }$$

Substitute (5), (6) into (4) to get the following equation.

11 $${K}_{s}=cot\beta tan\varphi +\frac{2{c}_{l}}{\gamma (L\mathrm{h}cos\beta +Ldsin\beta +hd)sin\beta }$$

If the vertical joint surface is a closed type and the tensile strength *σ*_*h*_ is too large to be ignored, *K*_*s*_ is expressed below.

12 $${K}_{s}=\frac{\gamma (Lhcos\beta +Ldsin\beta +hd)+{2\sigma }_{h}tan\beta }{\gamma (Lhcos\beta +Ldsin\beta +hd)tan\beta -{2\sigma }_{h}}tan\varphi +\frac{{2c}_{l}}{\gamma (Lhcos\beta +Ldsin\beta +hd)sin\beta -{2\sigma }_{h}cos\beta }$$

The formula for calculating the safety factor of slopes containing closed and open vertical joints is summarized as follows:

13 $$K_{s} = cot\beta tan\varphi + \frac{{c_{l} }}{{wsin\beta }}({\text{Open}}\;{\text{vertical}}\;{\text{joints}})$$

14 $$K_{s} = \frac{{w + \sigma _{h} tan\beta }}{{wtan\beta - \sigma _{h} }}tan\varphi + \frac{{c_{l} }}{{wsin\beta - \sigma _{h} cos\beta }}({\text{Closed}}\;{\text{vertical}}\;{\text{joints}})$$

It can be seen that there are many factors affecting the stability of the slope with vertical joints. The dip angle* β* and length *L* negatively correlate with the slope safety factor *Ks*. The length of vertical joints *h* and horizontal distance from the slope surface *d* negatively correlate with *Ks*. The tensile strength of the vertical joint surface *σ*_*h*_ positively correlates with *Ks*.

It is known from the above that the length of vertical joints, the *slope distance*, and the dip angle of the slip fracture surface are the main factors affecting the stability of the slope. Therefore, it is necessary to further investigate the stability of the jointed slope by combining it with numerical calculation.

Combined with the calculation results, there are extreme values of the influence on the stability of the slope, including shear failure inclination *β* at the back edge of loess slopes containing vertical joints, length *h* of vertical joints, and horizontal distance *d* from the slope surface. The slip characteristics of the slope with vertical joints are summarized as follows: the failure occurs along the vertical joints first in loess slopes with vertical joints, and the depth of pulling crack is related to the length of vertical joints; the maximum vertical pulling crack is up to 15 m; the optimal failure inclination of the back edge of pulling crack is 57°.

## Discussion

This paper proposes the sliding failure characteristics of loess slopes containing vertical joints based on the triaxial test and finite element numerical simulation method, which is of practical application value for predicting the sliding surface and sliding range of loess slopes containing vertical joints. The slope stability coefficient formula proposed in the paper can be used to assess the stability of loess slopes with vertical joints. When the number of joints is large, and the geological engineering conditions are complicated, the specific safety coefficient should be further calculated by combining with numerical simulation. The conclusions presented in the paper are based on the static state of the slope without considering the softening effect of water on the jointed surface and the effect of seismic inertia force, which will be further considered in the subsequent study.

## Conclusion

In this paper, the strength and failure characteristics of the vertical joint samples are obtained by unconfined compression tests and triaxial compression tests. Further, the influence of the length and dip angle of the joints on the stability of the loess slope containing vertical joints is analyzed through numerical calculation. Finally, the formula for the safety factor of the loess slope with vertical joints is arrived at by analytical calculation. The main contributions are as follows.

1. The soil mechanical experiments demonstrate that the joint affects the strength and failure rate of the samples. The unconfined compressive strength of the closed and open vertical joint samples reduces by 15.7% and 21.5%, respectively. The failure rate is raised by 2.28 times and 1.61 times, respectively.
2. The paper quantitatively evaluates the influence of the length of vertical joints and the *slope distance* on the slope stability and the overall sliding range. The safety factor of the slope decreases first and then increases with the increase of the joint length. When d = 10 m and h = 15 m, the degradation degree of the vertical joint to the slope with a height of 40 m and a slope of 45° reaches the peak.
3. The paper puts forward the sliding failure characteristics of loess slope with vertical joints. The tensile fracture depth no longer expands with the increase of joint length and the *slope distance* after the vertical tensile fracture along the vertical joint reaches 15 m. The shearing fracture of the back edge of the landslide is dominated by the failure inclination between 45° and 65°, and the optimal failure inclination is 57°.

## Data Availability

The datasets used and/or analyzed during the current study available from the corresponding author on reasonable request.
